# Community agency and empowerment—a need for new perspectives and deepened understanding

**DOI:** 10.1080/03009734.2018.1474303

**Published:** 2018-06-12

**Authors:** Mats Målqvist

**Affiliations:** International Maternal and Child Health, Department of Women’s and Children’s Health, Uppsala University, Uppsala, Sweden

**Keywords:** Agency, community, discourse analysis, empowerment, stakeholder

## Abstract

**Background:**

In an increasingly globalized and interlinked world it becomes ever more important to find strategies to prevent, detect, and respond to emerging public health threats. Local communities have a central role in this effort and need to be empowered and strengthened to be able to meet the challenge, and local knowledge and participation are key. This paper outlines a theoretical framework for community intervention dynamics and explores perceptions, priorities, and perspectives of stakeholders involved in community interventions.

**Methods:**

A deductive discourse analysis was performed based on the proposed theoretical framework consisting of three levels: intervention design, intervention delivery, and community agency. The setting was a workshop on community preparedness at Uppsala Health Summit 2017. Thirty-eight participants representing government officials, international organizations, and researchers as well as community implementers underwent a value exercise and were asked to prioritize good practices, challenges, and needed solutions to empower communities to meet emerging health threats.

**Results:**

The value exercise revealed a large variation in basic values among participants. Discussions mainly focused on intervention delivery and choice of methods. Need and allocation of resources at any level was not an issue. Despite being probed to take a deeper look at contextual factors and the underlying drivers of community engagement, participants scarcely mentioned and problematized community agency mechanisms.

**Conclusion:**

There is a need for new perspectives and a deepened reflection among decision-makers and public health implementers engaging at the local level to strengthen communities to face public health threats. A greater understanding and focus on contextual factors is needed which necessitates stronger interdisciplinary approaches.

## Introduction

We live in a rapidly changing world where global health issues have become interlinked and disease knows no borders. Emerging diseases like Ebola and Zika and challenges such as antibiotic resistance or obesity put high demands on both the international and local communities to have the ability to prevent, detect, and respond to these threats (1). Early warning and rapid response systems as well as health system strengthening are important components in this effort. But equally important are local communities’ ability and capacity to deal with local and global health challenges on the ground. Community resilience is a term often mentioned in this context, even if there is no unanimous definition of what this means (2). Patel et al. suggest that community resilience consists of nine elements: local knowledge, community networks and relationships, communication, health, governance and leadership, resources, economic investment, preparedness, and mental outlook (2). In summary, it is about being able to act, to have agency to develop and make the right choices—or, with another word, to be empowered. To strengthen this agency and to empower communities must be an integral part of public health efforts for all stakeholders.

We have the evidence of what needs to be done in relation to most public health issues. The problem is, however, how this knowledge can be implemented. Implementation science is a growing field which deals with this process of translating evidence into practice (3,4). Empowering communities to meet health challenges is one way to do this. Education and information are strategies with the underlying assumption that if only people know the benefits of a healthy living they will be empowered to change their lives. However, most public health practitioners know that this is rarely the case and that empowerment to change habits and promote health is influenced by a much more complex reality. Facilitating strategies like peer support, mentoring, awareness raising, and many more are needed to achieve change, and they require engagement with the community on the ground.

Another aspect of community intervention is context. Not all efforts are suitable at all places, and a one-size-fits-all approach seldom works. It is increasingly acknowledged that contextual knowledge is needed for successful implementation (5), and the request for anthropological and sociological methods is becoming stronger in global health (6). The SDGs emphasize the interdisciplinary nature of the challenges facing humankind, and disease outbreaks like the Ebola outbreak in western Africa in 2014–15 illustrate this vividly (7). But interdisciplinary methods require stakeholders to step outside their comfort zones and challenge their own perceptions and ways of working. There is a need for new perspectives and a deepened reflection.

The aim of this study was to investigate the discourse among decision-makers, health care planners, and other stakeholders working with communities in order to find out what the main concerns, priorities, and perceptions are when working to strengthen communities. This analysis would help to identify gaps in reasoning and pave the way for an interdisciplinary approach.

## Methods

### Theoretical framework

**Figure 1.**
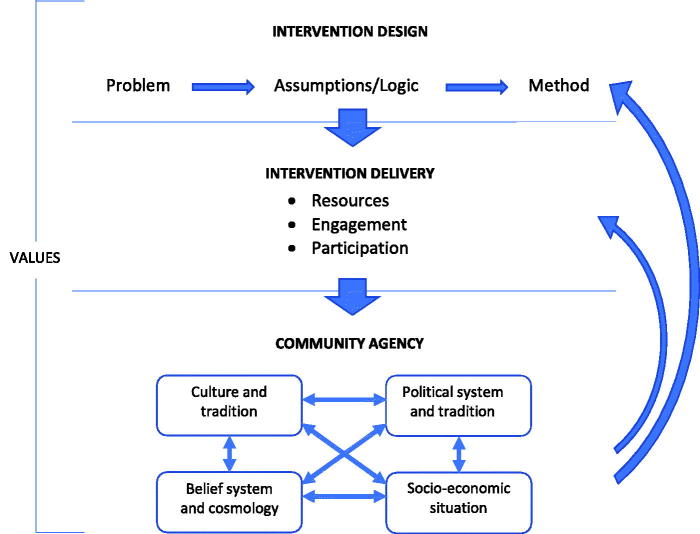
Theoretical framework.

A theoretical framework was developed by the author for analysis. This framework aims to capture the process of intervening at community level to strengthen preparedness and to improve health and social outcomes. The framework consists of three components: *intervention design*, *intervention delivery*, and *community agency* (Figure 1). These three components loosely correspond to the three components of the PARHiS framework that stipulates that evidence, facilitation, and context need to be considered when designing and implementing an intervention (8).

### Intervention design

Evidence guides intervention design and is the foundation of the problem statement that is being addressed. This means that both the focus of the intervention as well as the choice of implementation strategy is presumably based in evidence through research, needs assessments, or experience. The intervention design thus is a consequence of the problem at hand. There is a plethora of designs to choose from, and ideally these choices should be informed by the context in which they are to be implemented, as described by the arrows in the framework (Figure 1). In this framework, intervention design is divided into three sub-components: (1.1) the *problem* to be addressed; (1.2) the *assumptions* and values underpinning the process to develop an intervention; and (1.3) the *methods* chosen.

### Intervention delivery

The intervention delivery can but does not necessarily have to be a consequence of intervention design. When delivering an intervention to the community it can be done in different ways, with different levels of: (2.1) *resources*; (2.2) *engagement*; and (2.3) *participation*. Often the delivery is as important as the design. One major distinction often made when discussing intervention delivery is between a top-down and a bottom-up approach. A top-down approach usually implies that the intervention is delivered by an actor outside of the community, such as conducting training for community leaders, supplying food or materials, or distributing policy guidelines. A top-down delivery is most often the result of a top-down design, when needs and challenges have been identified without or with minimal involvement of the local community. A bottom-up approach is usually characterized as an intervention which originates in needs expressed by the community and with a delivery mode that has a high level of engagement and involvement of the community in which it is delivered. These two approaches are not mutually exclusive but can work in tandem or alternating. A top-down intervention design can thus be delivered in a bottom-up way, or the needs identified and raised by the community might require a top-down intervention delivery. Crucial components in intervention delivery, and what to a large extent decides whether an intervention will be delivered top-down or bottom-up, are therefore the level of engagement in the intervention by the intervener, the level of participation by the community in which it is delivered, and the level and source of resources added to the intervention. All these components will affect motivation and consequently outcome.

### Community agency

At the core of any community intervention is community agency, which is a crucial part of context. Agency is defined as the capability of achieving change or action when needed and is closely related to empowerment (9). There is a continuous debate within social sciences regarding the relationship between agency and social structure (10). To what extent are we free, both as individuals and as communities, to act in our own best interest, and how much is decided by the context we are living in? These perspectives interact, and many community interventions aim to increase empowerment by addressing structures, both external and internal. When intervening in the community there are four dimensions of community agency that need consideration: (3.1) the *local culture and tradition*; (3.2) the hegemonic *belief systems*; (3.3) the *political tradition and system*; and (3.4) the *socio-economic conditions*. These four elements interact and reinforce each other to further create and maintain the level of agency within a community.

Cultural practices are often mentioned in global health literature, with examples of harmful traditions that should be abolished or addressed. Culture is, however, so much more, since it is an important part of social capital. In relation of agency, culture has an important role in reproducing and guiding behaviors, setting the limits for conduct and appropriateness. Culture is closely linked to the hegemonic belief system. How we make sense of the world from an existential perspective provides explanatory models and justification to cultural norms and traditions. The hegemonic component of beliefs is an important aspect to take into consideration when interacting with communities and can often be what defines a certain community or sub-community.

Another important aspect of community agency is the political system: how things are governed and how decision-making power is distributed and delegated. This is not only influential on a practical level, but also reflected in thought patterns and mindsets. The political system and tradition dictate what is appropriate behavior when it comes to taking initiatives, who has a say, and what the limits of each individual’s actions are. It is thus closely linked to culture and belief systems. The fourth dimension in community agency is the socio-economic context. The level and distribution of resources limit or facilitate action and set the boundaries to what can be achieved. The level of resources is to a large extent decided by external factors, but both the generation and division of wealth are closely connected to the other three dimensions.

### Setting and participants

During Uppsala Health Summit 2017, an invitational conference with focus on emerging disease threats and how to tackle them with a One Health approach, 38 stakeholders were invited to a workshop on community preparedness. The conference was held in Uppsala, Sweden on 10–11 October 2017, and the workshop, which was part of the overall program, started at 10.30 a.m. and finished at 3.30 p.m. on the first day of the conference. Participants of the workshop had applied for the workshop, as an option of four different workshops at the same time, and had been purposively selected in order to secure a diversity of interests. The participants were all stakeholders with an interest in community interventions, ranging from high-level government officials to implementers in the field. Academia and private sector and non-governmental organizations were also represented. Participants originated and had their primary place of engagement in 12 different countries.

### Data collection

The workshop was divided into two sets with a 1-hour lunch break in between. Notes were taken by an independent observer during the full workshop. The first session began with two inspirational speakers focusing on community interventions and engagement in relation to infectious disease outbreaks. Examples and experiences from the Ebola outbreak in Sierra Leone in 2014–15 and the HIV epidemic in Swaziland were used to illustrate different aspects of how disease threats can be tackled at the community level. Thereafter participants were divided into six groups with 6–8 participants and asked to perform a value exercise as described below. The second session, after lunch, started with two presentations representing the government and international community perspective on the disease outbreaks in Sierra Leone and Swaziland. Participants were asked to identify good practices, challenges, and potential solutions on how to strengthen and engage with communities in order to encourage responsiveness and to record these on post-it notes. Post-it notes were later gathered for analysis.

A value exercise was introduced in order to capture underlying assumptions and values in relation to community engagement. Participants were given four different statements: (1) ‘Information changes behavior’; (2) ‘Resilience is measurable’; (3) ‘Cultural practices, even harmful, must be respected’; and (4) ‘Top-down approach is compatible with local participation’. They were then instructed to individually place markers along two crossing axes to grade if they thought the statement was accurate or not (yes–no) and whether it was a relevant issue to consider or discuss in relation to community engagement (relevant–not relevant). After individual placement, the participants were allowed to discuss within the group and re-place their markers, if desired.

### Data analysis

Final placement of markers in the value exercise was recorded before moving on to the next statement. Coordinates for all participants’ final placements were merged for analysis. Scatterplots and calculation of means was used for analysis. All analyses were performed in STATA/IC 12.1.

A deductive discourse analysis was applied to all gathered material (11), utilizing the theoretical framework described above. Both the presentations of the inspirational speakers and the following discussion were analyzed in order to detect differences in the discourse between the pre-arranged intentions of the organizers and the following discussions by participants.

### Ethics and consent

No ethical approval was sought for the study. No harm can be expected to arise from this study, and topics were clearly communicated beforehand. All workshop participants were made aware that results from discussion would be reported in different media. Anonymity has been preserved in the reporting.

## Results

**Figure 2.**
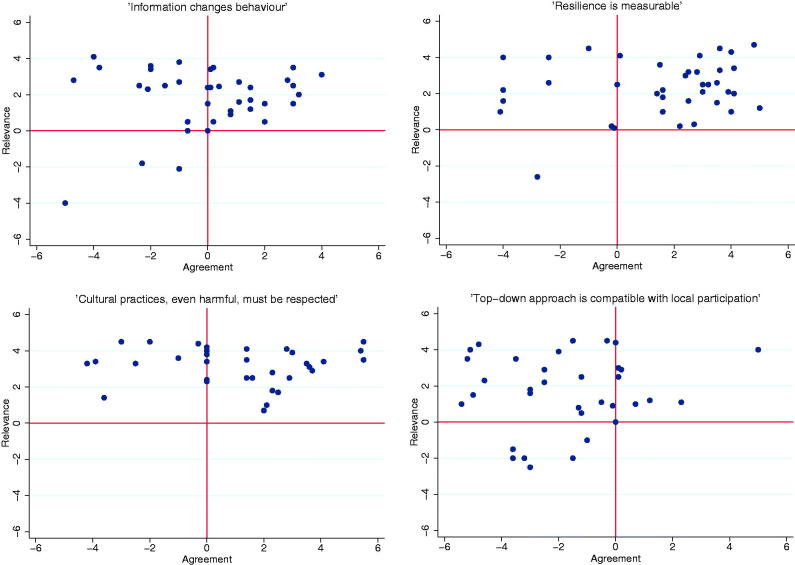
Scatterplots based on value exercise.

Visual results from the value exercise are displayed in Figure 2 (12). Overall there was a general consensus that the statements were relevant; the mean value on the *y*-axis ranged from 1.59 to 3.16. The statement ‘Information changes behavior’ displayed the largest division on agreement with a mean closest to zero (–0.05). For all statements, there was a wide range of agreement, and no consensus could be detected.

Based on the theoretical framework a discourse analysis was performed to assess topics discussed during the workshops. To set the scene the workshop was initiated by two inspirational speakers. First, Paul Richards, an anthropologist with a long-standing engagement in Sierra Leone, spoke about his experiences from the Ebola outbreak in the country in 2014. A lot of emphasis was put on cultural practices (3.1) and how efforts to contain and meet the disease threat were played out, especially focusing on the participation of community (2.3) (7). After Richards’s presentation Gunilla Hallonsten, a theologian who lived and conducted studies in Swaziland between 2000 and 2005, spoke about the HIV epidemic and the initial response from the end of the 1990s until 2007 when anti-retroviral treatment was made available at a large scale. Hallonsten put most of her emphasis on the cosmology and explanatory models (3.2) utilized when making sense of the new disease and its impact on community (13). The involvement of traditional healers, *sangomas*, illustrates how the local belief systems played a crucial role in meeting the challenge of HIV. Hallonsten also described the international community’s response and how international and local aid organizations dictated what should be done, with little participation and understanding of the community context (2.2, 2.3), while at the same time the organizations made a financial profit (2.1) (13).

After the value exercise described above, and a lunch break, two representatives of the international community perspective gave an account of their experiences of the Ebola outbreak in Sierra Leone and the HIV situation in Swaziland. Anders Nordström, who was the WHO representative in Sierra Leone at the time with the task to coordinate the response, described the process and measures taken by the international community. A lot of emphasis was put on how the intervention was delivered and how the interaction with the local community did not play out well in the beginning (2.3) despite heavy engagement from the international actors (2.2). One explanation stressed was the faulty assumptions made guiding the choice of interventions (1.2) and how the methods applied were not always appropriate and effective (1.3). Nordström concluded that it was not until the different actors changed their mode of delivery (2.2, 2.3) and took into account the local cultural practices and needs (3.1) that the response was successful. After this account from Sierra Leone, Samson Haumba, country director for the international organization University Research Collaboration (URC), shared his experiences from working with HIV prevention and response in Swaziland. Haumba focused almost exclusively on intervention delivery and the need for community participation (2.3). He also demonstrated how assumptions guide the choice of intervention methods (1.2) and how the methods used should take community knowledge into account (1.3).

The workshop participants were then divided into groups and asked to identify problems and challenges faced when aiming to strengthen communities’ ability to respond to disease threats. Participants were also asked to state possible solutions to these obstacles and identify good practices, as described in Methods section above. Each group wrote down their topics discussed on post-it notes and then shared their discussions with the other groups in an open forum. A summary of topics discussed is found in Table 1, where categorization according to the theoretical framework has been indicated. Most topics discussed related to intervention delivery and the level and mode of engage with the community (2.2) and how to relate to community participation (2.3). Notable is how the need and allocation of resources (2.1) was absent from all discussions. Financing mechanisms were discussed by one group, but then only in terms of the short-term of how to value local assets and initiatives, not how the actual lack of resources might put constraints on efforts. Corruption was also brought up by one group and then discussed in terms of the political context (3.3) and how systems should be put in place to counter corruption. The political dimension was also brought up in relation to the problem that local ownership is often poor and that there is a need for legislation against discriminatory behavior. Other aspects of community agency were brought up sparsely. One group mentioned that education of women and children might be a way to increase their socio-economic situation (3.4), and one group brought up the need to empower religious leaders to challenge existing belief systems (3.2). No references were made to culture and tradition (3.1) other than as general statements that there is a need to address culture. The problem definition (1.1) was not discussed in the groups, most likely because the topic, how to prevent, detect, and respond to emerging disease threats, was given beforehand. However, next to discussion of intervention delivery much focus was put on the underlying assumptions and values (1.2), and suggestions on different methods to be used (1.3) were given.

**Table 1.** Challenges and solutions of how to increase community empowerment and resilience discussed at a workshop at Uppsala Health Summit 2017.

Challenges	Solutions	Dimension
Mistrust at the time of crisis and how to build trust	Establish a functioning primary health care system that has sustainable funding and is accountable.	1.3
	Persuade and explain to the politicians that putting money into health care is a good investment for the future.	2.2
	Talk with the communities not to the community.	2.3
	Seek to understand risks, priorities, and challenges.	1.2
	Identify stakeholders.	1.2
	Find tools, processes, and platforms to engage stakeholders and community.	1.3
	Build networks, relationships, and social structures	2.3
	Empower religious leaders to address beliefs.	3.2
How to include marginalized groups	Engage and work with the community in order to explain why extra resources are allocated to certain groups while avoiding creation of more stigma.	2.3
	Find the leaders for the marginalized groups and design and carry out interventions together.	1.2, 2.3
	Raise awareness in the international society for less privileged religions.	1.2
How to connect and engage local and international actors	Better understanding of the bottom-up approach is needed.	1.2
	Use institutions and structures that already are in place.	1.3
	Engage anthropologists and local leaders.	1.3, 2.3
	Improve communication between institutions that are already in place.	2.2
	Follow up community interventions and give feed-back to community on what went well and what can be improved; this will also enhance and build trust.	2.2, 2.3
	Build knowledge about health challenges among non-health professionals.	1.1
Lack of women’s empowerment	Education can hopefully lead to strengthened economy and voice for women; it is also important to create incentives for girls to stay in school and for equal job opportunities.	3.4
	Advocate for undiscriminating legislation and its implementation.	3.3
	Challenge culture through socializing men.	3.1
	Recognize the gender gap and engage with women.	1.2
Lack of commitment, from the communities themselves, from the health system, lack of political will	Create political awareness and common will to build resilient health systems.	2.2
	Change the perception of CHWs from the role of volunteers into professionals with appropriate salaries.	1.2
	Organize community advisory boards with diverse members.	2.3
Lack of ownership of the problem at community level	Change volunteers’ perception and what they can achieve.	3.3
Short-term funding and lack of sustainability	Build a funding system where community-level applications are accepted.	1.3
	Local ownership offers opportunity for sustainability.	3.3
Corruption	Establish competent financial systems who track and follow up on funding to avoid corruption.	3.3
Poor communication within governance structures	Use trusted intermediaries.	2.3
	Feedback lessons learnt back into the community.	1.3
	Establish reporting systems.	1.3
	Utilize different communication strategies.	1.3
Cultural practices and barriers	Have tailored solutions.	1.3
	Engage in dialogue.	2.3
	Understand the rationale why people are doing certain things.	1.2
	Bring diverse knowledge systems to bear on problems.	1.3

## Discussion

The value exercise indicated that there was a wide spread of underlying ideas in relation to community intervention work. No consensus could be detected on the validity of the chosen statements, even if there was a general agreement that all statements were relevant. The latter might be a result of selection bias, since all participants can be assumed to have a special interest in community interventions. Overall, the value exercise was appreciated and discussions were lively, indicating a need to reflect on underlying values.

This deductive discourse analysis of a full-day workshop with an array of stakeholders involved in community action revealed a clear focus on intervention design and delivery rather than on community agency. The fundamental properties of community empowerment and resilience, the cultural, religious, political, and socio-economic aspects, were only used as a backdrop in discussions despite being the focus of the workshop theme. It is not understood why this was the case, but the lack of deepened understanding and problematizing of community agency in this diverse group of actors is a matter of concern. Despite being introduced to the area by two speakers focusing on the influence and importance of culture and tradition and the large impact of belief systems, participants tended to focus on the more immediate concerns of their own reality.

It is not possible to deduce the underlying reasons for this lack of complex and holistic understanding from the material at hand. One possible explanation might be found in the definition and connotations of development. It has been suggested that the general and historical view on development has focused more on quantitative measures than qualitative change, which risks resulting in misconceptions and a superficial approach to community interventions (14). Another explanation might be the sensational nature of the Ebola epidemic that was used as an example in the workshop. The high media interest and the exceptional situation pertaining in Sierra Leone at the time might over-shadow the possibility of a deeper analysis. On the other hand, the example of HIV that was also introduced in the workshop has a longer history and would lend itself better to reflection on practices and approaches used. To learn from mistakes and being able to show good examples should be easier with a more diverse history of prevention efforts. The different nature of HIV and Ebola should also be noted. Both being viral diseases that need community interventions to be curbed, they represent two endpoints on a spectrum, with Ebola being a quick and dramatic occurrence and HIV being a slow and silent opportunist with around seven years from contamination to disease expression. Despite the different strategies needed to tackle these two emerging disease threats, this was not reflected in the workshop discussions, which further emphasizes the lack on in-depth understanding of community intervention.

The results of this analysis also show that we still have a long way to go in the SDG agenda’s intention for a more holistic and interdisciplinary approach. As long as the main concerns among policy-makers, implementers, and researchers are on evidence and interventions and not on understanding and addressing the context, there is a great risk that we will continue to work in silos. The idea of interdisciplinarity is also not straight-forward, and more efforts are needed to better define both the concept and methodologies. Callard and Fitzgerald claim that ‘interdisciplinarity is a term that everyone invokes, but none understands’ (15, p. 4), which is a relevant reflection also in this context. However, in order not only to understand the fundamental mechanisms of a given context but also to be able to intervene and strengthen communities and build local agency we need to find new ways of addressing the problems and threats at hand and consider what needs to be done in a multidisciplinary way. How this should be done is still a great challenge. One suggested way to go is to look at the literature on community resilience development and learn from different strands of science. Resilience is a concept that in itself encompasses an holistic understanding, and Berkes and Ross suggest a combination of well-established socio-ecological models on resilience and the individual focus found in psychology and mental health disciplines (16). These two approaches can be combined with resilience as the common denominator, possibly leading to new perspectives and work methods with a more integrated and interdisciplinary onset. Another method to reap the benefits of working together across disciplines might be to adapt a ‘transdisciplinary’ approach by together finding new methods of scientific inquiry, borrowing methodology from different disciplines to address research questions in a new way. One such example can be found in the setup of a Master’s program in Health, Gender, and Religion at Stellenbosch University and University of KwaZulu-Natal in South Africa. The program utilized methods and traditions from public health, theology, law, and sociology of religion in order to reach deeper insights and perspectives on community health (17). These examples show the possibility and benefit of a deeper understanding and use of interdisciplinarity.

It can be argued that the stakeholders present at this workshop are not representative of the international stakeholder community at large. There can of course be some selection bias, but participants came from a large variety of backgrounds and had diverse experiences. All participants had also favored this workshop ahead of other options at the overall summit, indicating that they all had a special interest in the issues discussed. This would make the expectations on what to discuss higher. Another potential limitation of the study is related to the author being the workshop organizer. This introduces bias in the analysis and interpretation of results, since the analysis might be influenced by preconceived ideas. As a workshop organizer, you have a preset expectation of what will be discussed and how the sessions will play out. A final limitation is that the analysis was performed only by one person (the author), and it can be argued that this would compromise robustness. The analysis is, however, guided by a theoretical framework, and the interpretation is thus transparent, as compared to if an inductive approach had been chosen.

There is an apparent need for new perspectives and a deepened understanding of how to strengthen and empower communities. This deductive discourse analysis from a workshop with government officials, field implementers, and other stakeholders indicated a superficial understanding of the influence and mechanisms of contextual conditions when trying to strengthen communities to prevent, detect, and respond to emerging health threats. Much focus was put on intervention delivery, which is in one way reassuring since it displays an understanding that how we do things matters. At the same time, it is deeply problematic if the underlying structures are not addressed. If fundamental conditions of the dimensions of community agency are not dealt with, we risk only making cosmetic changes, not achieving true and long-lasting improvements.
